# Supplementary material for: metaAPA: a tool for integration of PolyA site predictions from single-cell and spatial transcriptomics
Source: Bioinform Adv. 2026 May 26;6(1):vbag147. doi: 10.1093/bioadv/vbag147 (PMC13259596; doi:10.1093/bioadv/vbag147)
Supplement: vbag147_Supplementary_Data [file vbag147_supplementary_data.pdf]

Supplementary Information

for "metaAPA: a tool for integration of PolyA site predictions from single-cell and spatial transcriptomics"

Qian Zhao, Magnus Rattray.

8 April 2026

—

- Supplementary Figure S1 Upset plots for other samples.
- Supplementary Figure S2 Integrated results of the two strategies at the *Rbis* gene locus.
- Supplementary Figure S3 Integrated results of the two strategies at the *Sox8* gene locus.
- Supplementary Figure S4. Scalability of metaAPA.
- Supplementary Figure S5. Extensibility of metaAPA.

—

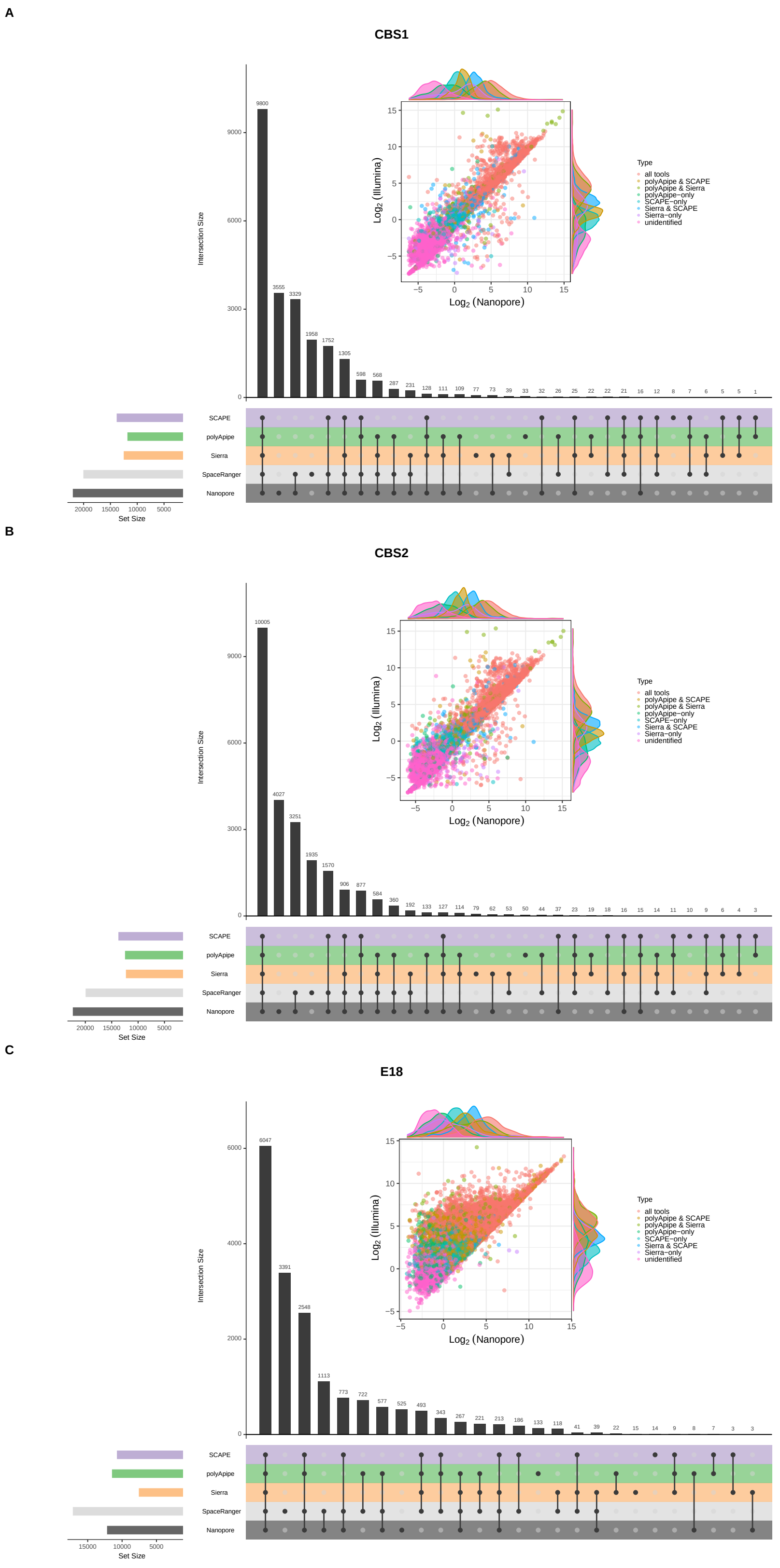

Figure S1. UpSet plots for other samples. (A) CBS1, (B) CBS2, and (C) E18.

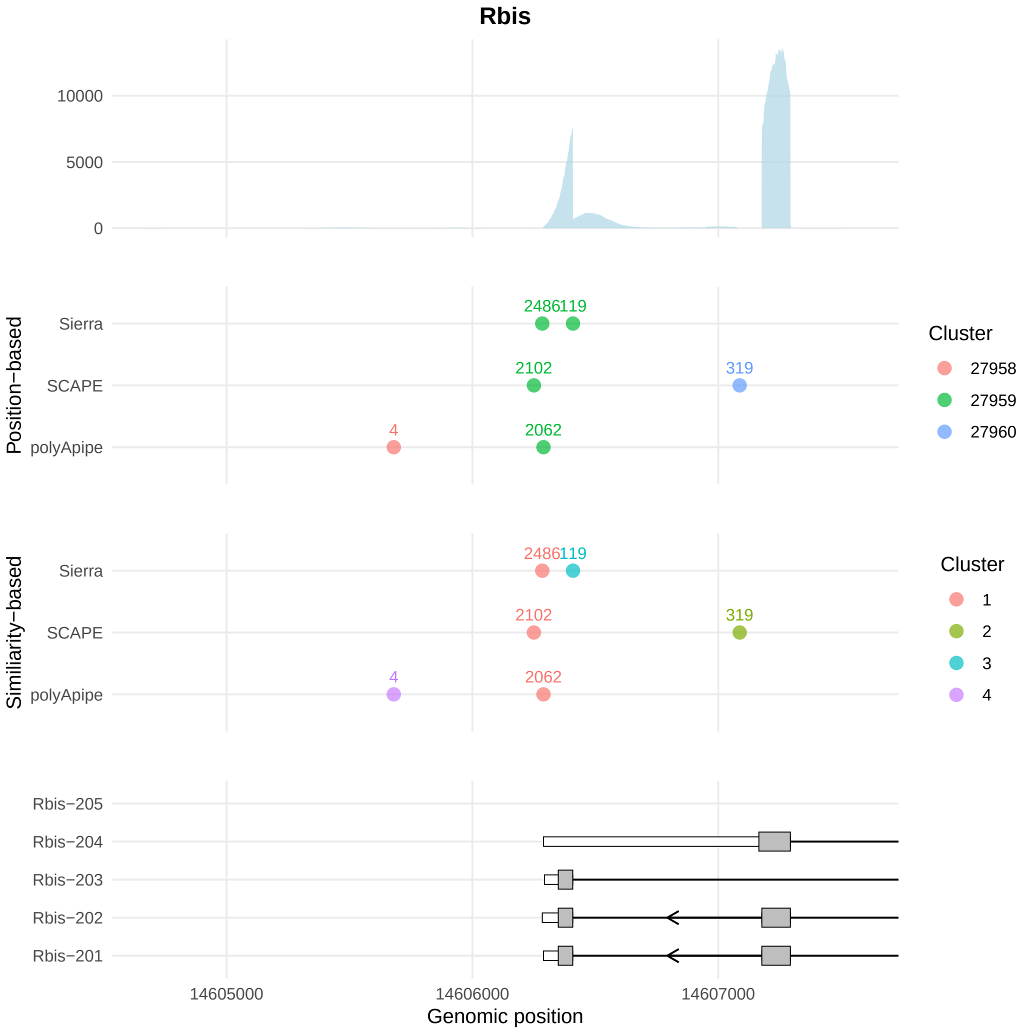

Figure S2. Integrated results of the two strategies at the *Rbis* gene locus. From top to bottom, the panels show the coverage plot, results from the position-based strategy, results from the similarity-based strategy, and genomic annotations.

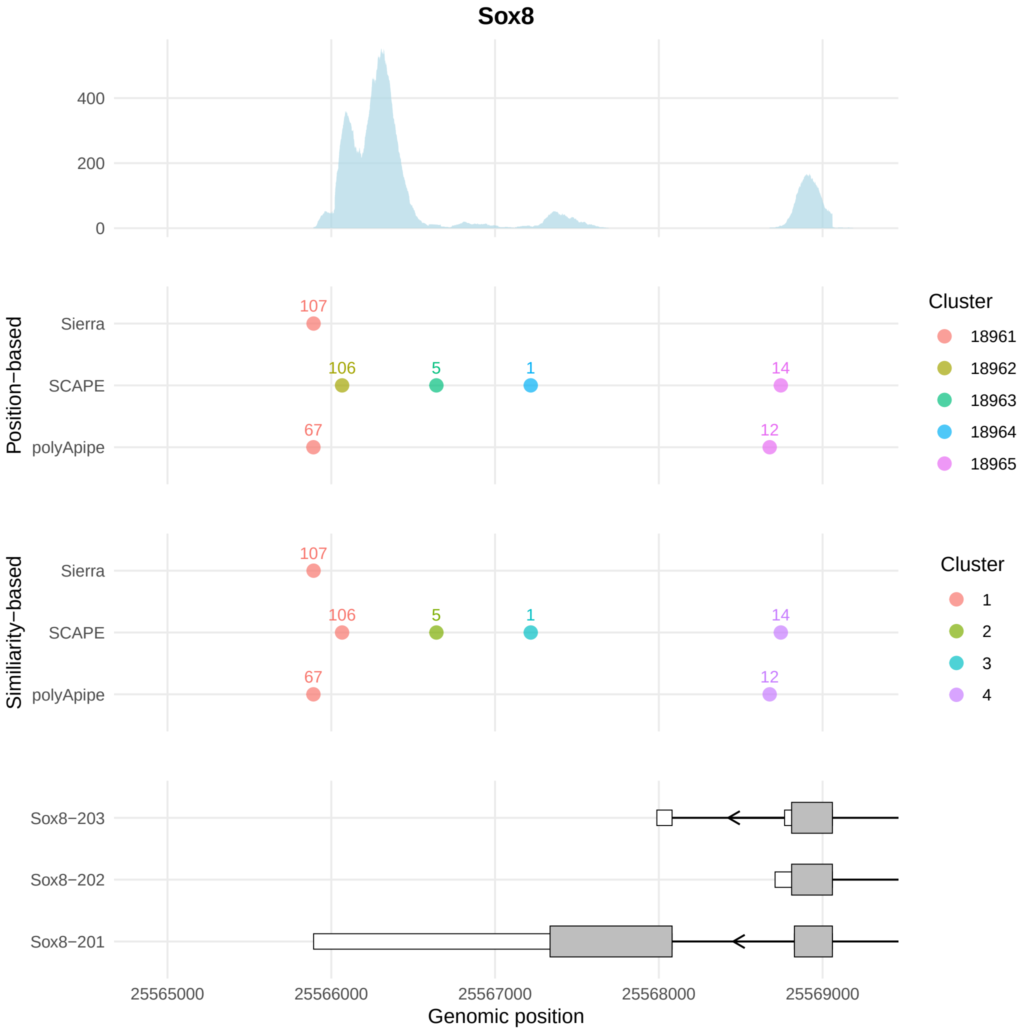

Figure S3. Integrated results of the two strategies at the *Sox8* gene locus. From top to bottom, the panels show the coverage plot, results from the position-based strategy, results from the similarity-based strategy, and genomic annotations.

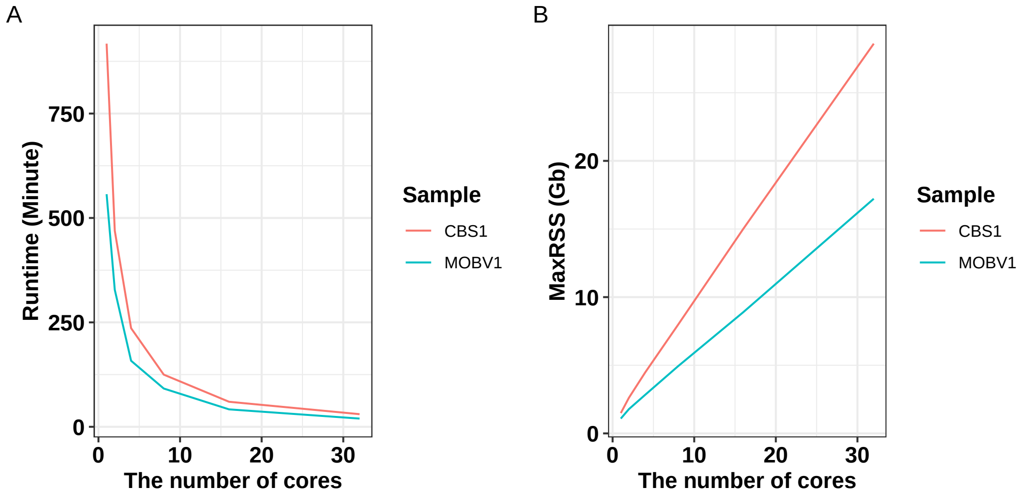

Figure S4. Scalability of metaAPA. (A) Runtime performance and (B) memory usage of metaAPA evaluated on the CBS1 dataset (2,560 spots and 217M reads) and MOBV1 dataset (918 spots and 254M reads). metaAPA was executed using the default configuration, which combines cosine distance with k-means clustering.

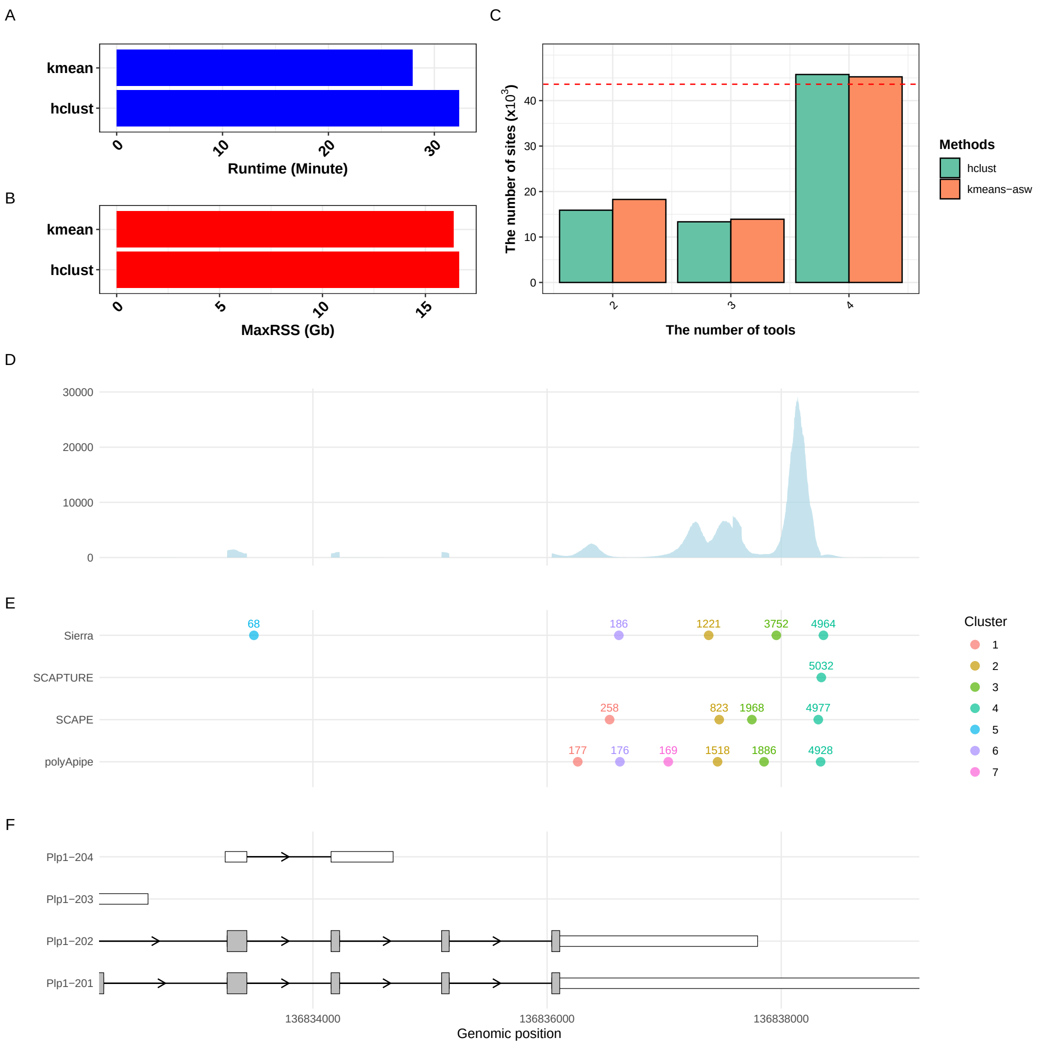

Figure S5. Extensibility of metaAPA. (A) Runtime and (B) memory usage of metaAPA on the MOBV1 dataset (918 spots and 254M reads). metaAPA was executed using the cosine distance with k-means and hclust clustering. (C) The number of high-confidence sites obtained using the similarity-based integration strategy after incorporating the new method SCAPTURE. The red dashed line indicates the high-confidence site threshold. Here, x-axis represents different confidence levels, or equivalently, the number of tools supporting a given site. A value of 4 indicates sites supported by all four tools, while a value of 3 indicates sites supported by three tools. Sites supported by three or more tools are considered high-confidence sites. (D). Coverage profile of the *Plp1* gene in the MOBV1 sample. (E). Integration results from four methods. (F). Genome annotation of *Plp1* gene.
